# Serum antiphospholipid antibody status may not be associated with the pregnancy outcomes of patients undergoing in vitro fertilization

**DOI:** 10.1097/MD.0000000000029146

**Published:** 2022-03-25

**Authors:** Xiao-Fang Tan, Li Xu, Ting-Ting Li, Yan-Ting Wu, Wei-Wei Ma, Jia-Yi Ding, Hong-Li Dong

**Affiliations:** a *Department of Reproductive Medicine Centre, Affiliated Maternity & Child Health Care Hospital of Nantong University, Nantong, Jiangsu province, China,*; b *Scientific Education Section and Department of Child Healthcare, Affiliated Maternity & Child Health Care Hospital of Nantong University, Nantong, Jiangsu province, China.*

**Keywords:** antiphospholipid antibodies, in vitro fertilization, meta-analysis

## Abstract

**Background:**

Antiphospholipid syndrome (APS) is an autoimmune disease that is associated with recurrent pregnancy loss. It is still controversial whether the presence of antiphospholipid antibodies (aPL) in the serum of patients with in vitro fertilization-embryo transfer (IVF-ET) has a negative effect on the outcomes. In view of the discrepancies, a meta-analysis of the published data was performed to explore the relationship of aPL and IVF-ET outcomes.

**Methods:**

We searched for all published articles indexed in PubMed, Web of Science, and Cochrane Library, which were retrieved up to April, 2021. A total of 921 studies were yielded, of which 6 finally met the inclusion criteria. We carried out the meta-analysis by pooling results of these studies with Review Manager 5.3 software. The effect index was measured with 95% confidence intervals (CIs) of the relative risks (RRs).

**Results:**

Six eligible studies were included in this meta-analysis, involving 3214 patients. Our results showed that positive aPL was not associated with decreased clinical pregnancy rate (RR 0.97; 95% CI 0.91-1.04). There was no correlation between positive aPL and increased miscarriage risk (RR 1.22; 95% CI 0.94-1.58). Only 5 of the 6 studies referred to live birth rate, but still no association was found between them (RR 0.95; 95% CI 0.81-1.11).

**Conclusions:**

The results showed that the presence of positive aPL neither decreased clinical pregnancy rate and live birth rate, nor increased miscarriage rate in women undergoing IVF, which is differed from the opinion of clinical practice. More prospective studies with high quality and larger sample size are needed to evaluate the relationship between positive aPL and outcomes of IVF-ET.

## 1. Introduction

Antiphospholipid antibodies (aPL) is a group of autoantibodies reported to be related to risk for recurrent pregnancy loss (RPL), fetal growth restriction, pre-eclampsia, and thrombosis.^[1,2]^ Therefore, aPL screening is recommended for the diagnosis and treatment of RPL by the European Society of Human Reproduction.^[3]^ Recently, more and more interesting are focused on the relationship between aPL and recurrent implantation failure (RIF) in women undergoing in vitro fertilization-embryo transfer (IVF-ET), because there are some similarities between RPL and RIF in terms of the presence of antiphospholipid antibodies.^[4]^ However, the results on the relationship between aPL status and embryo implantation is uncertain.

A previous meta-analysis published in 2000 reported that positive aPL was not significantly associated with IVF-ET pregnancy outcomes.^[5]^ However, the result was challenged by the American Society for Reproductive Immunology, and was thought to set a dangerous precedent.^[6]^ Thereafter, a number of prospective cohort studies have been carried out in order to find out the correlation between positive aPL and IVF-ET outcomes, which were more evident than retrospective studies. Moreover, these results were still controversial.

Considering the conflicting results, we collected all the prospective studies published so far on the relationship between positive aPL status and IVF outcomes to perform a meta-analysis aiming to assess the effect size of aPL status and determine the extent of heterogeneity between studies in terms of the strength of the association with the goal of providing evidence for clinical practice.

## 2. Materials and methods

### 2.1. Search strategy, inclusion, and exclusion criteria

Several relevant databases, including PubMed, Web of Science, and Cochrane Library were searched up to April 2021. The following key words with different combinations were used during the literature search: antiphospholipid antibodies, anticardiolipin antibodies, antiphospholipid, anti-beta 2 glycoprotein I antibodies, lupus anticoagulant, anti-phosphatidylserineantibodies, anti-phosphatidylinositol antibodies, antiphosphatidic acid antibodies, anti-phosphatidylethanolamine antibodies, infertility, in vitro fertilization, IVF, intracytoplasmic sperm injection, in vitro fertilization and embryo transfer, assisted reproduction technology, assisted conception, gamete intrafallopian transfer, zygote intrafallopian transfer, tubal embryo transfer, frozen embryo transfer, pregnancy, pregnancy outcome, outcome, implantation, and miscarriage. In addition, a manual search of relevant reference articles was also performed for the meta-analysis.

Eligible studies were included if they met all of the following criteria: prospective cohort study; infertile women undergoing IVF; cases with positive aPL and controls with negative aPL; a definition of positive aPL as one or more abnormal results for anticardiolipin, anti-phosphatidylserine antibodies, anti-phosphatidyl ethanolamine antibodies, anti-phosphatidyl choline antibodies, anti-phosphatidylinositol antibodies, anti-phosphatidyl glycerol antibodies, β2 glycoprotein I, and lupus anticoagulant in the peripheral blood analysis; and IVF outcomes focusing on the clinical pregnancy rate, miscarriage rate, and live birth rate. Clinical pregnancy was defined as the observation of a gestational sac on ultrasound 4 to 5 weeks after embryo transfer. Miscarriage was defined as a clinical pregnancy that was lost before 20 weeks of gestation. The live birth rate was defined as the number of live births divided by the number of infertile women undergoing IVF.

Studies with the following features were not taken into consideration: case-control or retrospective studies; review articles, letters, comments, and reports; studies with incompleted data for statistical analysis; and studies involving the treatment of antiphospholipid antibody positivity.

### 2.2. Data extraction and quality assessment

The following data were extracted from the included studies based on specific standards: the name of the first author, year of publication, country of origin, type of study, ages of the subjects; number of cases with positive and negative aPL, and outcomes of IVF-ET.

Literature quality assessment was performed in accordance with the Newcastle Ottawa scale, as recommended by the Agency for Healthcare Research and Quality. Any disagreements were settled by discussion between the authors or decided by another expert.

### 2.3. Statistical analysis

The data were extracted from the 6 possible studies on the IVF outcomes of infertile women with positive aPL or negative aPL who had never received aPL related treatments. Meta-analysis was carried out using Review Manager 5.3 software (Cochrane Collaboration), and the effect index was measured with 95% confidence intervals (CIs) of the relative risks (RRs). Chi-square tests based on the *Q* statistic^[7]^ and *I*^2^ statistics^[8]^ were used to assess the heterogeneity test. The random effects model was used to combine the data for the heterogeneous outcomes (*P* < .05 or *I*^2^ ≥ 50%); otherwise, the fixed effects model was used. The Egger test was performed to assess publication bias (Figures S1-S3, Supplemental Digital Content, http://links.lww.com/MD2/A964). *P* values < .05 were considered statistically significant.

## 3. Results

### 3.1. Characteristics of the studies

**Figure 1.**
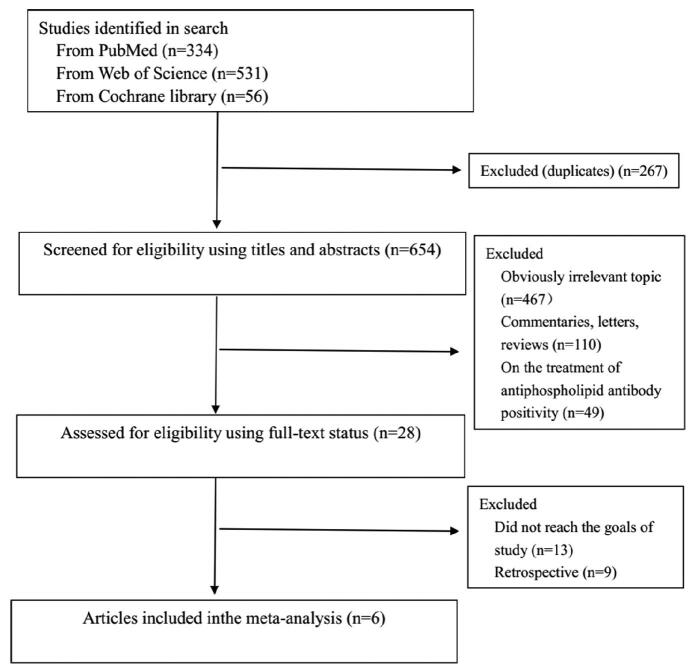
Flow diagram of study selection process.

The literature screening process of the studies is shown in Fig. 1. Relevant papers were obtained from the databases (PubMed 334, Web of Science 531, Cochrane Library 56) and manual search. A total of 921 papers were obtained from the original screening. Six hundred fifty four articles remained after deleting duplicated papers. Since reading the titles and abstracts, 626 articles were excluded because of irrelevant topic, comment, letter, reviews, and on treatment of aPL, 22 studies were then excluded (13 did not reach the goals of study, 9 were retrospective studies) after screening the full text. At last, 6 prospective studies from 28 left papers were included in the present analysis^[9-14]^ (Table 1). The general information and characteristics of the 6 eligible studies were listed in Table 2. The studies were carried out in the United States, China, United Kingdom, South Korea, and France, separately. All of these papers are written in English.

**Table 1:** Characteristics of the included studies assessing antiphospholipid antibodies in women undergoing IVF.

**Author**	**Year**	**Participants**	**Antiphospholipid antibodies**	**Control**	**Outcomes**
Birdsall et al	1996	No more than 3 previous IVF cycles (n = 240)	ACA, APs	aPL negative	CPR, MR, LBR
Denis et al	1997	Patients attempting to conceive through IVF (n = 793)	ACA, APs, APE, APC, API, APG, aPA	aPL negative	CPR, MR, LBR
Chilcott et al	2000	Women referred for IVF treatment (n = 380)	ACA, LA, β2GPI, APs	aPL negative	CPR, MR, LBR
Sanmarco et al	2007	Women with at least 3 unsuccessful IVF attempts (n = 101)	ACA, LA, β2GPI, APE	aPL negative	CPR, MR, LBR
Chen et al	2017	Women with accepted indication for IVF or ICSI (n = 1507)	ACA, β2GPI	aPL negative	CPR, MR, LBR
Hong et al	2018	Infertile women undergoing their first IVF (n = 193)	ACA, LA, β2GPI	aPL negative	CPR, MR

β2GPI = β2-glycoprotein, ACA = anti-cardiolipin, aPA = anti-phosphatidic acid, APC = anti-phosphatidyl choline, APE = anti-phosphatidyl ethanolamine, APG = anti-phosphatidyl glycerol, API = antiphosphatidyl inositol, aPL = anti-phospholipid antibody, APs = anti-phosphatidylserine, CPR = clinical pregnancy rate, LA = lupus anticoagulant, LBR = live birth rate, MR = miscarriage rate.

**Table 2:** Methodological quality of prospective cohort studies included in the meta-analysis.

**First author**	**Selection of the unexposed cohort**	**Ascertainment of exposure**	**Outcome of interest not present at start of study**	**Control for important factor or additional factor**	**Outcome assessment**	**Follow-up long enough for outcomes to occur**	**Adequacy of follow up of cohorts**
Birdsall et al	Yes	Yes	Yes	Yes	Yes	Yes	Yes
Denis et al	Yes	Yes	Yes	Yes	Yes	Yes	Yes
Chilcott et al	Yes	Yes	Yes	Yes	Yes	Yes	Yes
Sanmarco et al	Yes	Yes	Yes	Yes	Yes	Yes	Yes
Chen et al	Yes	Yes	Yes	Yes	Yes	Yes	Yes
Hong et al	Yes	Yes	Yes	Yes	Yes	Yes	Yes

### 3.2. Meta-analysis

The 6 eligible studies included in the meta-analysis involved 3214 patients undergoing IVF-ET. One thousand three hundred ten cases had at least 1 positive aPL, and 1904 women with negative aPL were as controls. The average rates of clinical pregnancy, miscarriage, and live birth were 56.6%, 14.3%, and 57.1%, respectively, in the aPL-positive patients, while 49.5%, 11.5%, and 53.8%, respectively in aPL-negative patients.

**Figure 2.**
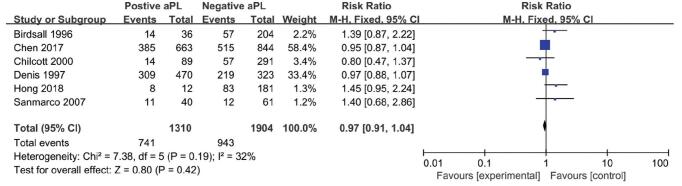
Forest plot of prospective cohort studies on antiphospholipid antibodies and the clinical pregnancy rate.

**Figure 3.**
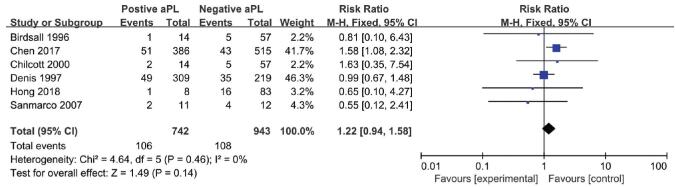
Forest plot of prospective cohort studies on antiphospholipid antibodies and the miscarriage rate.

**Figure 4.**
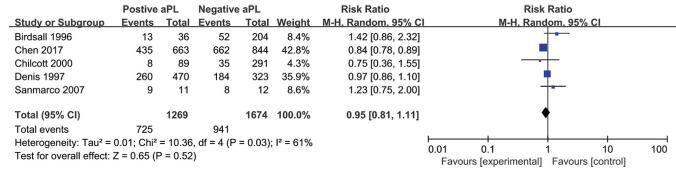
Forest plot of prospective cohort studies on the antiphospholipid antibodies and live births rate.

The RR suggested a relationship between aPL status and the IVF-ET outcomes were shown in the Figs. 2-4: clinical pregnancy (ranging from 0.80 to 1.45, Fig. 2), miscarriage (ranging from 0.55 to 1.63, Fig. 3), and live birth (ranging from 0.75 to 1.42, Fig. 4). However, none of the studies reflected a significant impact of aPL on the outcomes mentioned above. Therefore, as assessed by the clinical pregnancy rate in all 6 studies, there was no association between aPL abnormalities and all the negative IVF-ET outcomes (clinical pregnancy rate: RR 0.97, 95% CI 0.91-1.04; miscarriage rate: RR 1.22; 95% CI 0.94-1.58; live birth rate: RR 0.95, 95% CI 0.81-1.11) after pooling these prospective studies.

## 4. Discussion

aPL are a group of autoantibodies that target membrane phospholipids and phospholipid-binding proteins, and aPL comprise > 15 types of antibodies: the IgG, IgM, and IgA types of anticardiolipin, anti-phosphatidylethanolamine, anti-phos-phatidylserine, anti-phosphatidylinositol, anti-phosphatidylglycerol, anti-β2-glycoprotein-I, and lupus anticoagulant.^[15]^ It is reported that aPL stimulated endothelial cells, monocytes, and platelet activation, which lead to excessive production of tissue factors and thromboxane A2 to form thrombi.^[16]^ In 2006, the Sydney International Society for Thrombo hemostasis revised antiphospholipid syndrome (APS) clinical and laboratory standards. To diagnose APS, both clinical and laboratory indicators must be met. It is indirectly pointed out that APS is related to RPL, eclampsia, and other pathological pregnancies. Single positive aPL is only one of the conditions for the diagnosis of APS, and is not necessarily pathogeny of pregnancy.^[17]^

Positive aPL is more prevalent in infertility, recurrent spontaneous abortion (RSA), preeclampsia, and intrauterine growth restriction among women.^[18]^ Comprehensive reflection and meta-analysis showed a positive relationship between positive aPL and recurrent miscarriage in patients,^[19]^ and heparin and aspirin treatment are efficacious for patients who have experienced RSA. Some studies suggested that recurrent miscarriages and recurrent implantation failure shared certain similarities with APS. The presence of aPL or APS could be related to RSA and implantation failure.^[4,20]^

In 2008, the American Society for Reproductive Medicine declared that positive aPL had no negative effect on IVF outcomes.^[21]^ However, other studies have suggested that aPL reduced the pregnancy rate in women undergoing IVF-ET.^[13,22]^ In 2015, the Deepa “non-standardized” obstetrical antiphospholipid syndrome diagnostic criteria considered as including failed of embryo transfer ≥ 2 times as one of the non-standardized clinical criteria.^[23]^

The present meta-analysis results were consistent with the findings from the previous meta-analysis, showing that there was no significant correlation between positive aPL and IVF-ET outcomes, such as the miscarriage and live birth rates. Only the prospective studies were included in present meta-analysis, thus our results were more reliable compared with the meta-analysis of 2000 with the retrospective and prospective cohort studies.^[5]^

Nevertheless, because of the variety aPL, there were difference of antibodies studied among the included studies in this metaanalysis. The majority of the studies tested only 3 aPL among the various aPL family, therefore, the actual aPL prevalence would be underestimated. Furthermore, the occurrence of positive aPL was reported vary from 4.2% to 30.4% in women with IVF failure.^[14]^ The possible reasons may refer to the use of different antibodypanels, the selection of different patient groups, and the lack of evaluation standard. Moreover, aPL tests should be verified at least 6 weeks after the first test to exclude false positivity. In this meta-analysis, antibodies mentioned were limited in the included studies, therefore it is necessary to expand the aPL types in future studies, or classify each antibody into a subgroup for further analysis. In addition, there are complex influence factors for IVFET outcomes. And the infertility etiologies are various among the studies, IVF outcomes may be affected by factors such as male infertility and endometriosis. Besides, the discrepancy of regions/countries, ethnicities, the body mass indexes of involved cases, and the ovarian stimulation protocols used in the different studies may also impact the quality of results. Because of these limitations, the authors tried to improve the quality by including the studies according to the criteria seriously.

Based on the current research evidence, there is no significant correlation between positive aPL and IVF-ET outcomes. Therefore, routine screening of aPL may not be necessary for patients undergoing IVF-ET. However, the European Society for Human Reproduction and Embryo states that patients who have experienced frequent pregnancy loss should be screened for aPL.^[3]^ Many scholars believe that recurrent pregnancy loss and recurrent implantation failure are different stages of the same pathological progress. Similar to pregnancy loss, the normal endometrial receptivity was hampered due to blood could not flow to the endometrium and placenta, which ultimately lead to implantation failure. Moreover, the live birth rate in patients with positive aPL may be increased by aspirin treatment.^[24]^ Krivonos et al^[25]^ also found that women with aPL might have insufficient decidualization of the endometrium for embryo implantation, which was clinically manifested by implantation failure. In 2013, a systematic review and meta-analysis revealed that the pregnancy outcomes for women with RIF can be improved with injections of low-molecular-weight heparin, which enhances endometrial receptivity and trophoblast invasion.^[26]^ However, it is necessary to further study the relationship between RIF and positive aPL in IVF-ET patients.

In conclusion, this meta-analysis suggests that aPL status is not associated with IVF outcomes. Nevertheless, further investigations with a larger sample size, prospective, double-blind, placebo-controlled and randomized controlled studies are essential to further verify the relationship between aPL status and IVF outcomes.

## Acknowledgments

The authors would like to acknowledge Nantong Science and Technology Bureau for funding this study.

## Author contributions

Hong-Li Dong conceived and designed the research; Hong-Li Dong, Xiao-Fang Tan, Li Xu, Ting-Ting Li, Yan-Ting Wu, Wei-Wei Ma, Jia-Yi Ding collected the data; Xiao-Fang Tan performed the data analysis and drafted this paper. Hong-Li Dong critically revised the manuscript. Hong-Li Dong had primary responsibility for final content. All authors read and approved the final manuscript.

**Conceptualization:** Hongli Dong.

**Data curation:** Xiao-Fang Tan, Ting-Ting Li, Yan-Ting Wu, Wei-Wei Ma, Jia-Yi Ding, Hongli Dong.

**Formal analysis:** Xiao-Fang Tan, Li Xu, Ting-Ting Li, Yan-Ting Wu, Wei-Wei Ma, Jia-Yi Ding, Hongli Dong.

**Funding acquisition:** Hongli Dong.

**Investigation:** Xiao-Fang Tan, Li Xu, Ting-Ting Li, Yan-Ting Wu, Wei-Wei Ma, Jia-Yi Ding, Hongli Dong.

**Methodology:** Xiao-Fang Tan, Li Xu, Ting-Ting Li, Yan-Ting Wu, Wei-Wei Ma, Jia-Yi Ding, Hongli Dong.

**Project administration:** Li Xu, Jia-Yi Ding, Hongli Dong.

**Resources:** Hongli Dong.

**Software:** Xiao-Fang Tan, Hongli Dong.

**Supervision:** Hongli Dong.

**Writing** - **original draft:** Xiao-Fang Tan.

**Writing** - **review & editing:** Hongli Dong.

## Supplementary Material

SUPPLEMENTARY MATERIAL
