# Supplementary figures and images for: Serum antiphospholipid antibody status may not be associated with the pregnancy outcomes of patients undergoing in vitro fertilization
Source: Medicine (Baltimore). 2022 Mar 25;101(12):e29146. doi: 10.1097/MD.0000000000029146 (PMC11319311; doi:10.1097/MD.0000000000029146)

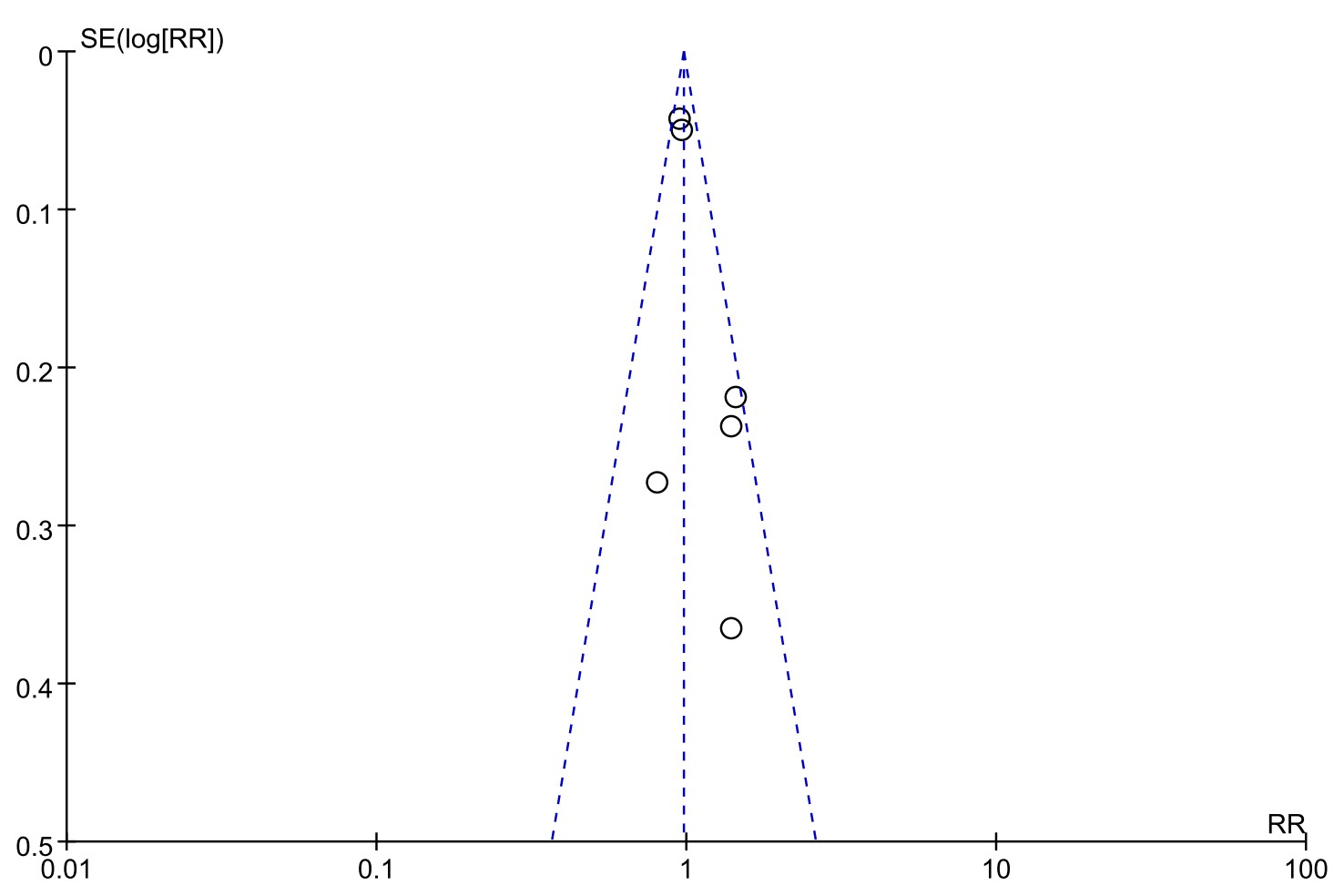

Supplement: SUPPLEMENTARY MATERIAL [file medi-101-e29146-s001.jpg]

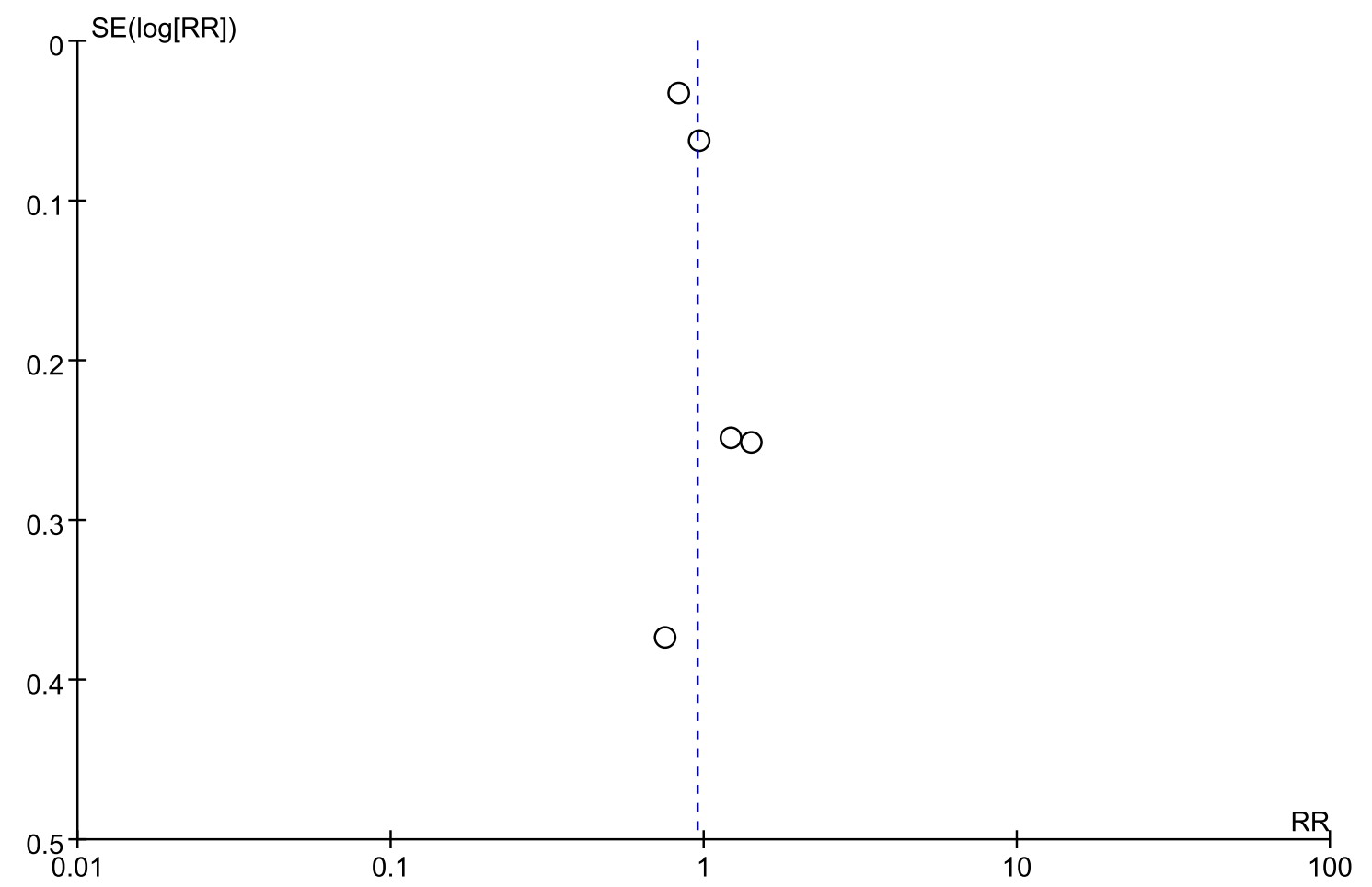

Supplement: SUPPLEMENTARY MATERIAL [file medi-101-e29146-s002.jpg]

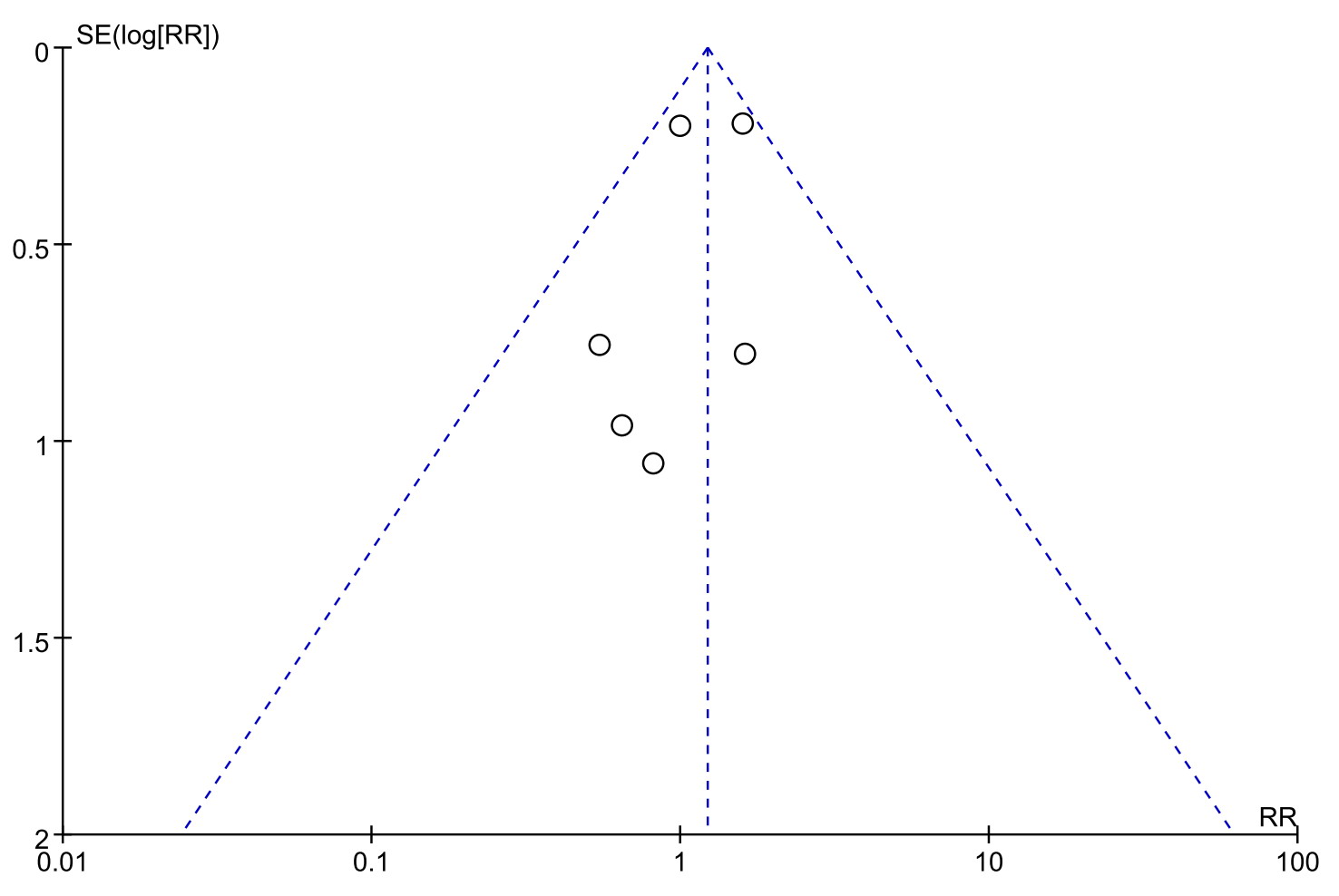

Supplement: SUPPLEMENTARY MATERIAL [file medi-101-e29146-s003.jpg]
